# Supplementary material for: Pd Nanoparticles Loaded on Cu Nanoplate Sensor for Ultrasensitive Detection of Dopamine
Source: Sensors (Basel). 2024 Sep 2;24(17):5702. doi: 10.3390/s24175702 (PMC11397903; doi:10.3390/s24175702)
Supplement: Supplementary file 1 [file sensors-24-05702-s001.zip › sensors-3105186-supplementary.pdf]

Supporting Information

Pd Nanoparticles Loaded on Cu Nanoplate Sensor for Ultrasensitive Detection of Dopamine

Haihu Tan 1, Xuan Zhang 1, Jinpu Xie 1, Zengmin Tang 2, Sijia Tang 2, Lijian Xu 2,* and Pingping Yang 2,*

1 College of Packaging and Materials Engineering, Hunan University of Technology, Zhuzhou 412007, China; tanhaihu2020@hut.edu.cn (H.T.); 18856765173@163.com (J.X.)

2 College of Life Science and Chemistry, Hunan University of Technology, Zhuzhou 412007, China

* Correspondence: xlj235@163.com (L.X.); yangpingping202211@163.com (P.Y.);

Tel.: +86-0731-22182980

Table of Contents:

1. Supplementary Figures

Fig. S1. The structure of BPEI.

Fig. S2. The UV-visible spectrum of Cu nanoplates suspension and Pd/Cu NPTs suspension

Fig. S3. (A) and (B) SEM images of Cu nanoplates.

Fig. S4. The size distribution of Cu nanoplates.

Fig. S5. STEM (A) and TEM−EDS mapping images of Cu (B) and Pd (C).

Fig. S6. The size distribution of Pd nanoparticles on Cu nanoplates of Pd/Cu NPTs.

Fig. S7. The lattice distance of HR−TEM characterization from Cu nanoplates.

Fig. S8. TEM images of Pd/Cu NPTs.

Fig. S9. STEM (A) and TEM-EDS mapping images of Cu (B) and Pd (C).

Fig. S10. DPVs of Pd/Cu NPTs/GCE at different pH (6.0, 6.4, 6.8, 7.2, 7.6) in 0.2 M PBS containing 5.0 mM DA.

Fig. S11. DPV-response of bare GCE in the presence/absence of DA.

Fig. S12. TEM images of Pd/Cu NPTs−20.

Fig. S13. CVs of GCE (A, B), Cu NPTs/GCE (C, D), Pd/Cu NPTs/GCE (E, F) in 5×10^{-3} M $K_3[Fe(CN)_6]$ solution (containing 0.1 M KCl) at different scan rates.

Table S1. The current changes when interfering substances was added.

Table S2. Determination of DA in human serum samples (n = 3).

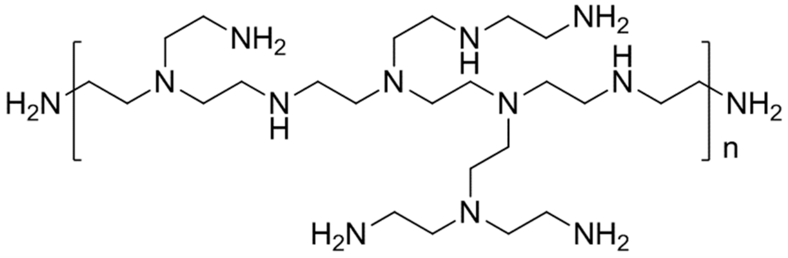

Fig. S1. The structure of BPEI.

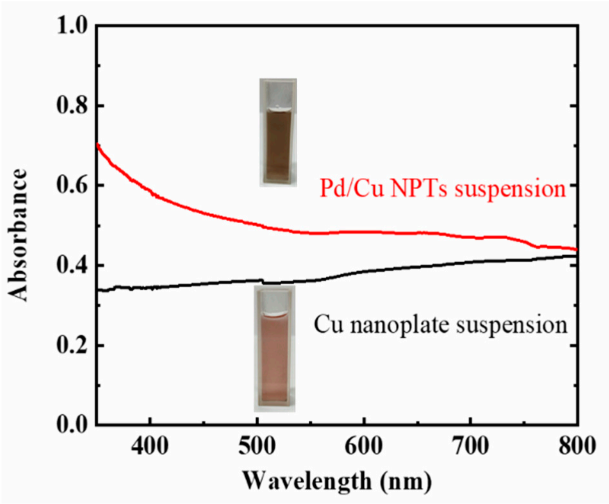

Fig. S2. The UV-visible spectrum of Cu nanoplates suspension and Pd/Cu NPTs suspension

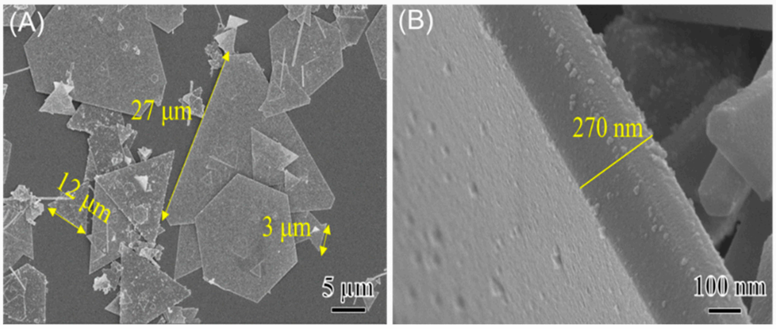

Fig. S3. SEM images (A, B) of Cu nanoplates.

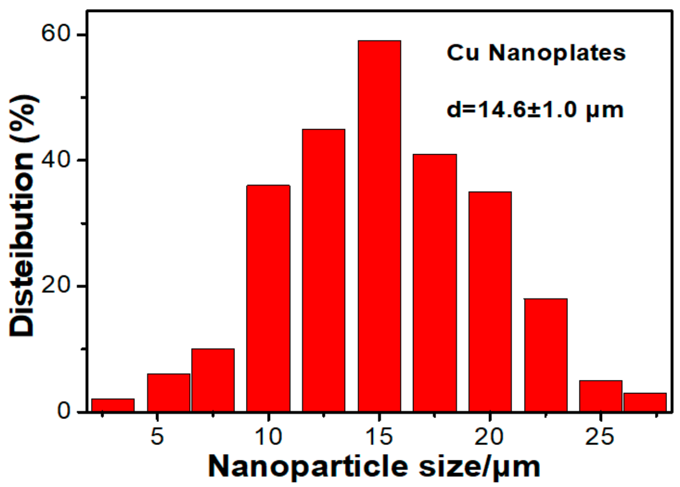

Fig. S4. The size distribution of Cu nanoplates.

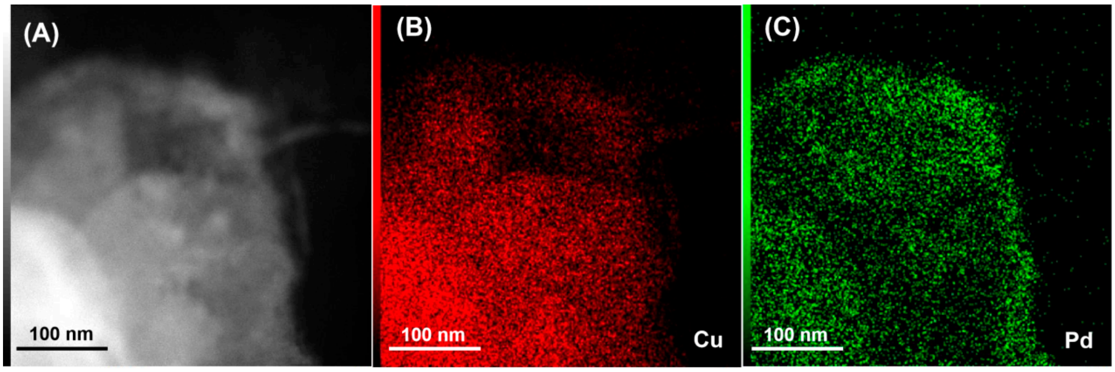

Fig. S5. STEM (A) and TEM-EDS mapping images of Cu (B) and Pd (C).

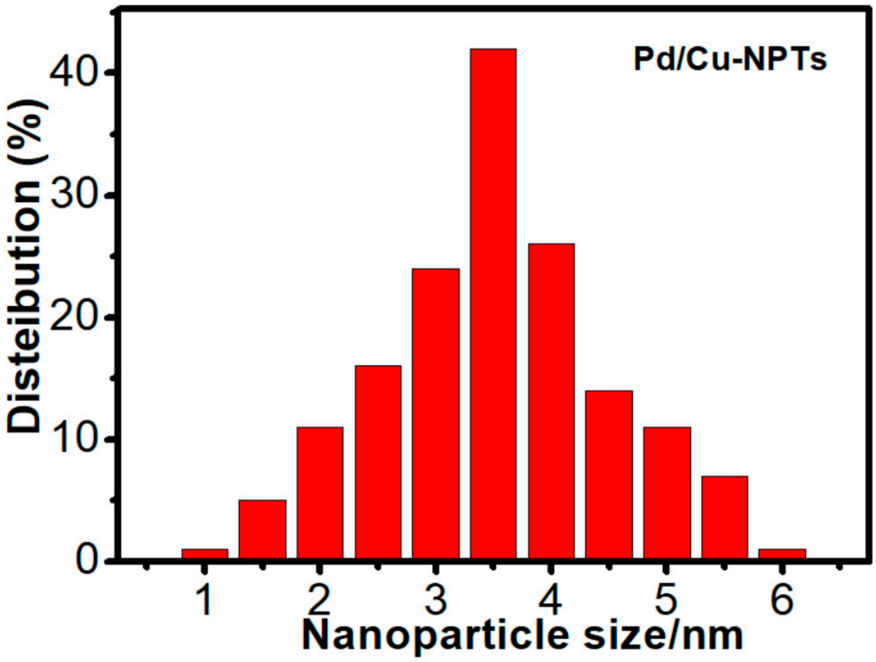

Fig. S6. The size distribution of Pd nanoparticles on Cu nanoplates of Pd/Cu NPTs.

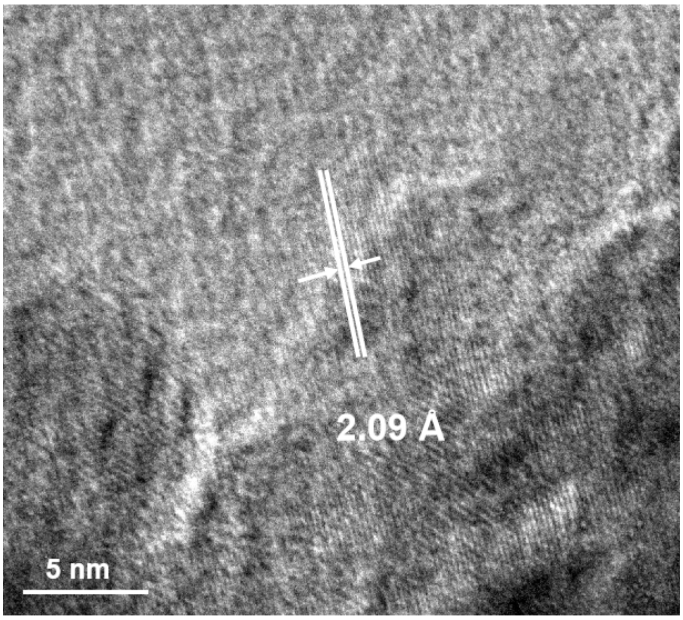

Fig. S7. The lattice distance of HR-TEM characterization from Cu nanoplates.

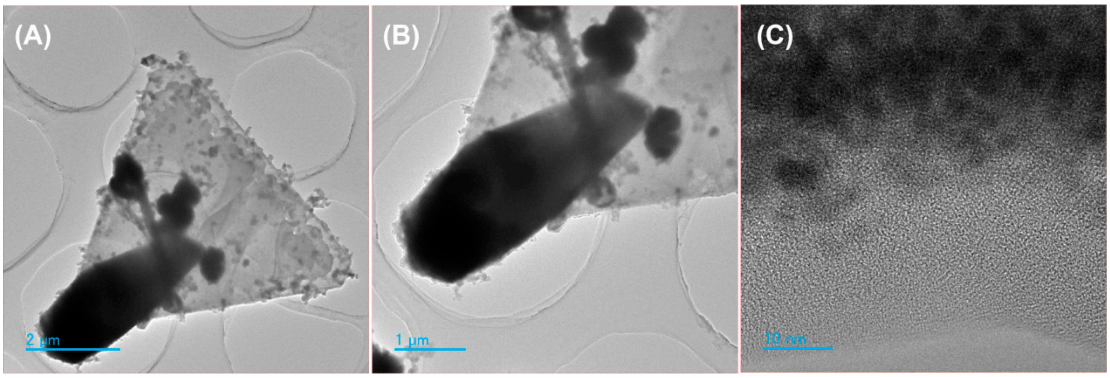

Fig. S8. TEM images of Pd/Cu NPTs.

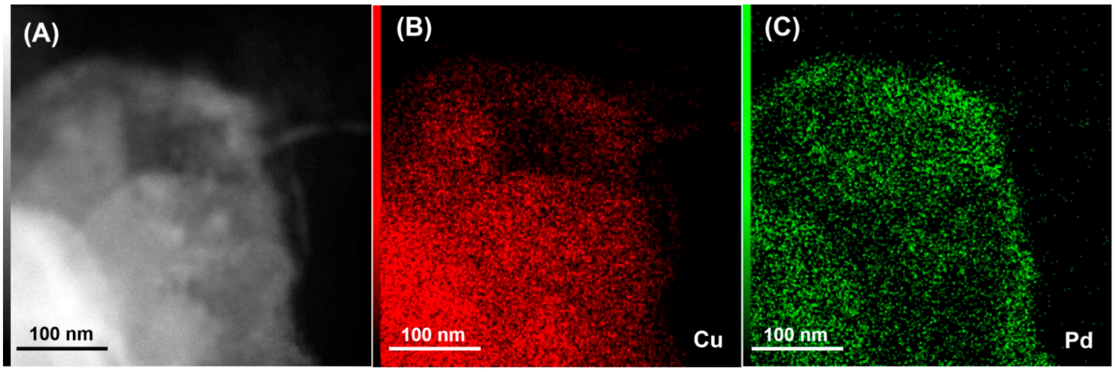

Fig. S9. STEM (A) and TEM-EDS mapping images of Cu (B) and Pd (C) of Pd/Cu NPTs.

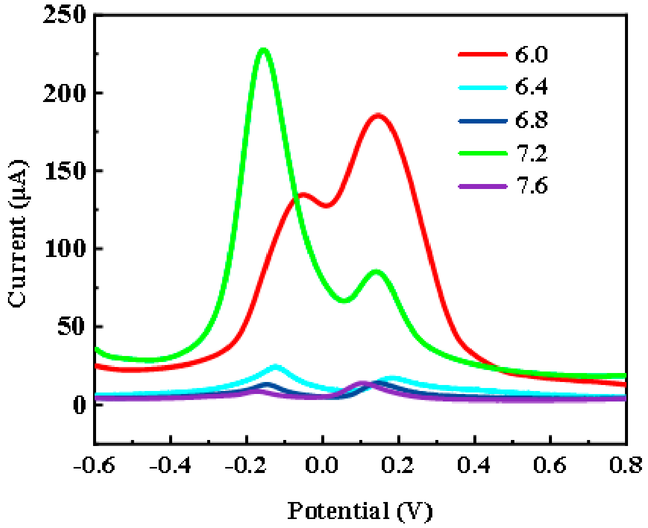

Fig. S10. DPVs of Pd/Cu NPTs/GCE at different pH (6.0, 6.4, 6.8, 7.2, 7.6) in 0.2 M PBS containing 5.0 mM DA.

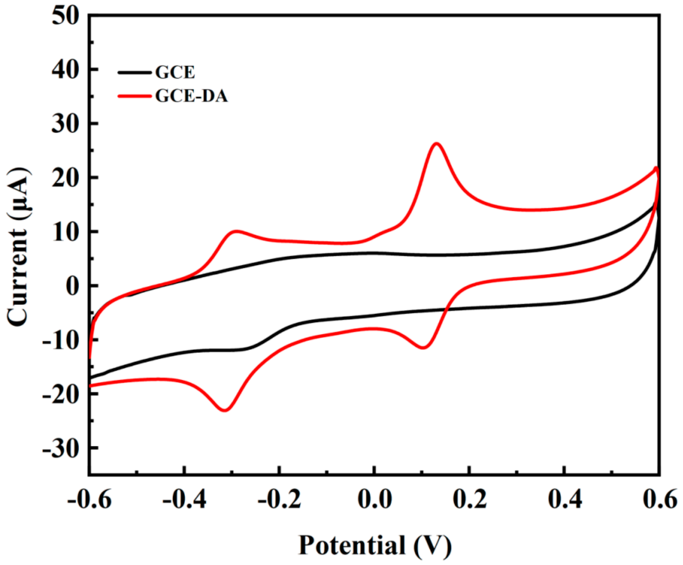

Fig. S11. DPV-response of bare GCE in the presence/absence of DA

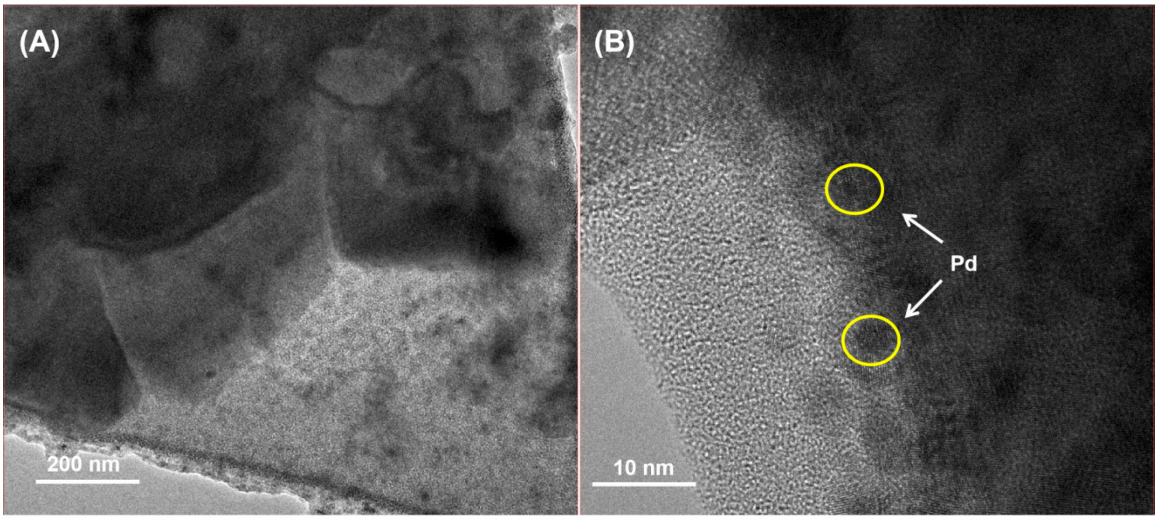

Fig. S12. TEM images of Pd/Cu NPTs-20.

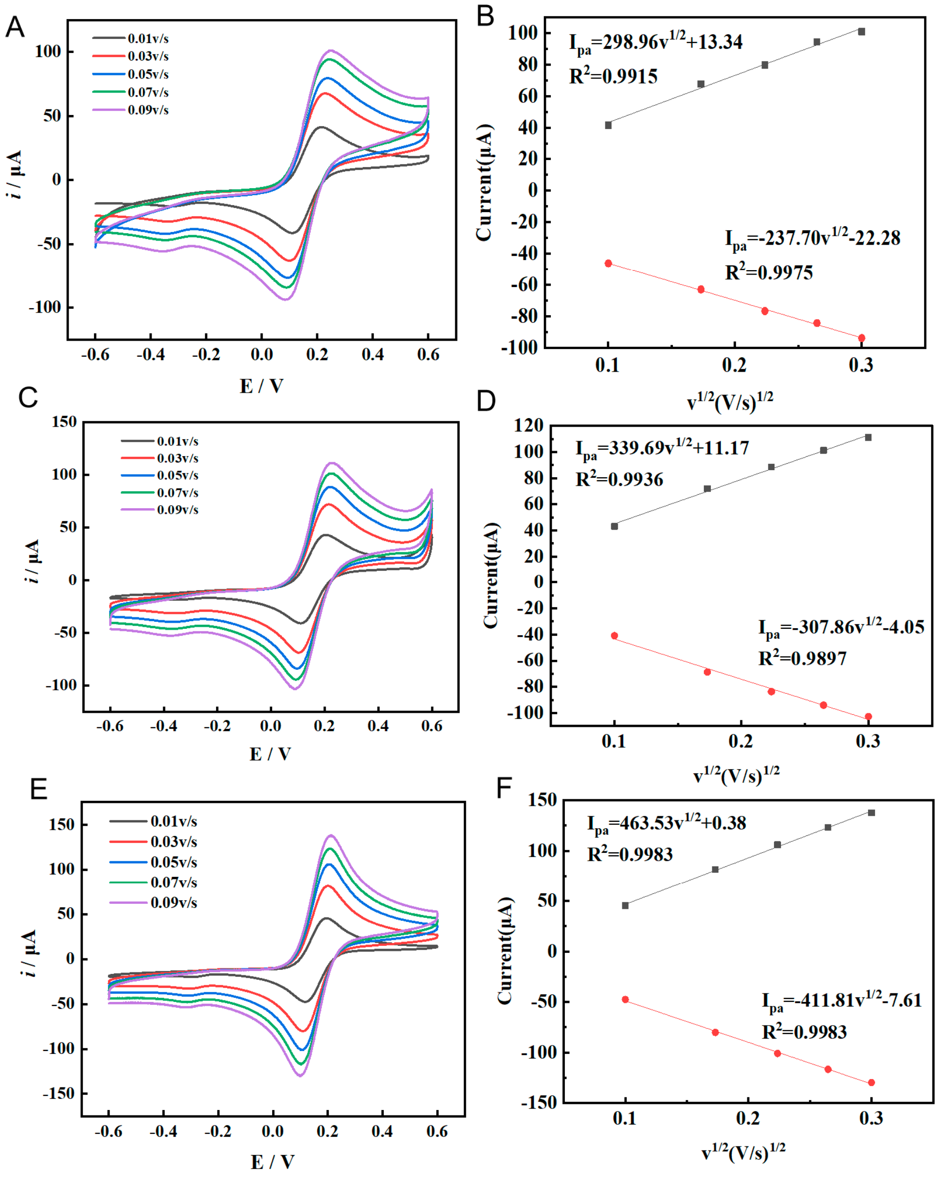

Fig. S13. CVs of GCE (A-B), Cu NPTs/GCE (C-D), Pd/Cu NPTs/GCE (E-F) in 5×10^{-3} M $K_3[Fe(CN)_6]$ solution (containing 0.1 M KCl) at different scan rates.

Table S1. The current changes when interfering substances was added.

	GLU	NaCl	UA	AA
before-addition current (μA)	18.5	17.2	16.3	15.1
after-addition current (μA)	17.2	16.3	15.1	17.3
The change in current (μA)	-1.3	-0.9	-1.2	+2.2

Table S2. Determination of DA in human serum samples (n=3).

	Added (g/L)	Found （g/L）	RSD (%)	Recovery (%)
1	13.14	12.51	-3.3	99.8
2	27.63	27.5	-1.31	97.5
3	48.62	48.77	1.2	100
